# Reconsidering angiography as the initial approach for post‐PCNL hematuria

**DOI:** 10.1002/ccr3.8383

**Published:** 2023-12-28

**Authors:** Niki Tadayon, Saleh Shahsavari, Dorsa Alijanzadeh

**Affiliations:** ^1^ Department of General and Vascular Surgery Shohada Tajrish Hospital Shahid Beheshti University of Medical Sciences (SBMU) Tehran Iran; ^2^ Department of Surgery Shohada Tajrish Hospital Shahid Beheshti University of Medical Sciences (SBMU) Tehran Iran; ^3^ School of Medicine Shahid Beheshti University of Medical Sciences(SBMU) Tehran Iran

Dear Editor,

We read the article by Salimi et al. on the endovascular management of post‐PCNL vascular injuries with great interest.^1^ In their study, the authors successfully diagnosed and treated post‐percutaneous nephrolithotomy (PCNL) hematuria in fourteen patients using angiography and subsequent embolization. They reported a 100% success rate, with ten patients having pseudoaneurysm (PA), four having arteriovenous fistula (AVF), and one having both subscapular hematoma and PA.^1^ The authors concluded that angiography is a safe and effective method for diagnosing etiology and treating post‐PCNL hemorrhage.

While we acknowledge the significance of the interventions and outcomes presented in this article, we believe further clarification on certain aspects is necessary. First, it is crucial to understand the criteria used to determine the necessity of an invasive procedure such as angiography and coil embolization. The authors did not specify the threshold for significant hematuria that prompted the invasive intervention. Factors such as the number of units of packed red blood cells transfused, the presence of shock, or the duration of gross hematuria following the index PCNL procedure should be elucidated to provide a clearer context for their approach.

Additionally, the authors attributed gross hematuria to PA or AVF in all cases. However, it is important to acknowledge that post‐PCNL hematuria can have other causes and treatments, including infection, as reported by Dhangar and colleagues.^2^ Even in cases with vascular etiologies such as PA, other noninvasive alternatives, such as administration of tranexamic acid, have been reported by Kumar et al.^3^ and Feng et al.^4^ as effective solutions.

Considering the diversity in etiology and management options for post‐PCNL hematuria, we propose that utilizing noninvasive investigations, such as computerized tomography (CT) angiogram, before proceeding to angiography, an invasive procedure, would be a reasonable approach. This could help in better patient selection for invasive procedures, potentially reducing the risk and cost associated with unnecessary interventions. This point would be clearer with a larger patient cohort.

In conclusion, despite the benefits and precision of angiography, we suggest that it might be better for physicians to consider noninvasive utilities like CT angiograms as the first step of evaluation and also have a risk assessment for ordering invasive investigation until clear clinical and laboratory data indicate post‐PCNL vascular injury needs angioembolization.

## AUTHOR CONTRIBUTIONS

**Niki Tadayon:** Conceptualization; supervision; writing – original draft; writing – review and editing. **Dorsa Alijanzadeh:** Writing – review and editing. **Saleh Shahsavari:** Writing – review and editing.

## FUNDING INFORMATION

None declared.

## CONFLICT OF INTEREST STATEMENT

All the authors declare no conflict of interest.

## Data Availability

The letter has no new or unreviewed data.
